# A rare case of Rosai–Dorfman disease presenting with cardiac tamponade

**DOI:** 10.1093/ehjci/jey232

**Published:** 2019-01-21

**Authors:** Jason M Tarkin, Virginia Wolstenholme, Michael Sheaff, Mark Westwood, Charlotte Manisty

**Affiliations:** 1 Department of Cardiovascular Medicine, National Heart & Lung Institute, Imperial College London, Hammersmith Campus, DuCane Road, London, UK; 2 Division of Cardiovascular Medicine, University of Cambridge, Addenbrooke's Hospital, Hills Road, Cambridge, UK; 3 Department of Oncology, St Bartholomew's Hospital, W Smithfield, London, UK; 4 Department of Cellular Pathology, Royal London Hospital, Whitechapel Road, London, UK; 5 Department of Cardiology, Barts Heart Centre, W Smithfield, London, UK; 6 Institute of Cardiovascular Science, University College London, Chenies Mews, London, UK

**Figure:**
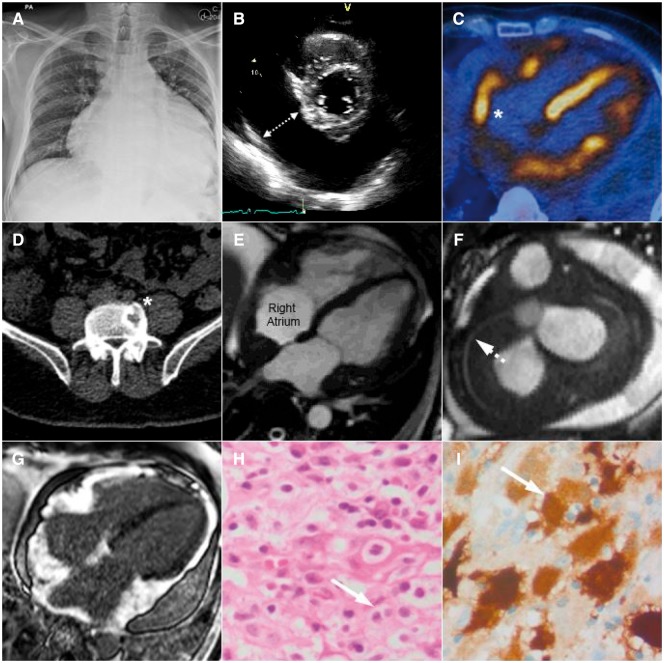


A 40-year-old man presented to hospital with exertional dyspnoea. A large pericardial effusion was identified by (*Panel A*) chest X-ray and (*Panel B*) transthoracic echocardiography, with echocardiographic features of early cardiac tamponade, including diastolic collapse of the right ventricular free wall (Supplementary data online, Video S1). He had been treated with vinblastine and prednisolone for multi-system Langerhan’s histiocytosis 6 years prior, with perianal cutaneous lesions, bone metastases, and cranial diabetes insipidus arising from pituitary gland involvement. To investigate for disease recurrence, ^18^F-fluorodeoxyglucose positron emission tomography-computed tomography was performed following emergency pericardiocentesis, which revealed (*Panel C*) abnormal tracer uptake in the heart, particularly the right atrium (asterisk) and (*Panel D*) lytic bone lesions in the spine (asterisk). Cardiac magnetic resonance imaging demonstrated an epicardial mass encasing the heart, the right coronary artery (dashed arrow), and superior vena cava; (*Panel E*) four-chamber and (*Panel F*) short-axis steady-state free precession cine images. The mass had high-native T1 values, was vascular on first pass perfusion imaging (Supplementary data online, Video S2), and strongly enhanced following administration of (*Panel G*) gadolinium contrast, suggestive of a solid tumour. Pericardial biopsy demonstrated a diffuse polymorphic cellular infiltrate, consisting of lymphocytes, plasma cells, and numerous large multi-nucleated cells with ill-defined pale cytoplasm. These abnormal cells displayed (*Panel H*) emperipolesis (arrow) and stained positive for CD163 and (*Panel I*) S100 (arrow), but not CD1a or Langerin. The underlying diagnosis was Rosai–Dorfman disease; a rare idiopathic non-clonal proliferative histiocytic disorder that involves the heart in <1% of cases. Cardiac involvement in Rosai-Dorfman disease can present as epicardial disease, with pulmonary arterial involvement, or as an intracardiac mass.

## Funding

J.M.T. was supported by the Wellcome Trust [211100/Z/18/Z] and the National Institute for Health Research. C.M. was supported directly and indirectly by the National Institute for Health Research University College London Hospitals Biomedical Research Centre, and the National Institute for Health Research Barts Biomedical Research Centre. 

## Supplementary Material

jey232_Supp

